# Accuracy, acceptability and feasibility of photography for use in trachoma surveys: a mixed methods study in Tanzania

**DOI:** 10.1093/inthealth/ihad111

**Published:** 2023-12-23

**Authors:** Donal Bisanzio, Robert Butcher, Valérian Turbé, Kenji Matsumoto, Chaitra Dinesh, Patrick Massae, Michael Dejene, Cristina Jimenez, Colin Macleod, Einoti Matayan, Caleb Mpyet, Alex Pavluck, Martha Idalí Saboyá-Díaz, Fentahun Tadesse, Sandra Liliana Talero, Anthony W Solomon, Jeremiah Ngondi, George Kabona, Cecilia Uisso, Alistidia Simon, Upendo Mwingira, Emma M Harding-Esch

**Affiliations:** RTI International, Washington, DC, USA; Clinical Research Department, London School of Hygiene & Tropical Medicine, London, UK; Department of Medicine, University College London, London, UK; Clinical Research Department, London School of Hygiene & Tropical Medicine, London, UK; Clinical Research Department, London School of Hygiene & Tropical Medicine, London, UK; Department of Ophthalmology, Kilimanjaro Christian Medical Centre, Moshi, Tanzania; Sightsavers, Addis Ababa, Ethiopia; Sightsavers International, Haywards Health, UK; Clinical Research Department, London School of Hygiene & Tropical Medicine, London, UK; Department of Ophthalmology, Kilimanjaro Christian Medical University College, Moshi, Tanzania; Department of Ophthalmology, University of Jos, Jos, Nigeria; Sightsavers Nigeria Country Office, Kaduna, Nigeria; RTI International, Washington, DC, USA; Communicable Diseases Prevention, Control, and Elimination Department, Pan American Health Organization, Washington, DC, USA; Federal Ministry of Health, Addis Ababa, Ethiopia; Escuela Superior de Oftalmología, Instituto Barraquer de América, Bogotá, Colombia; Global Neglected Tropical Diseases Programme, World Health Organization, Geneva, Switzerland; RTI International, Washington, DC, USA; Neglected Tropical Disease Control Program, Ministry of Health, Dodoma, Tanzania; Neglected Tropical Disease Control Program, Ministry of Health, Dodoma, Tanzania; Neglected Tropical Disease Control Program, Ministry of Health, Dodoma, Tanzania; RTI International, Washington, DC, USA; Neglected Tropical Disease Control Program, Ministry of Health, Dodoma, Tanzania; National Institute for Medical Research, Dar-es-Salaam, Tanzania; Clinical Research Department, London School of Hygiene & Tropical Medicine, London, UK

**Keywords:** acceptability, *Chlamydia trachomatis*, feasibility, photography, Tanzania, trachoma

## Abstract

**Background:**

Photography could be used to train individuals to diagnose trachomatous inflammation—follicular (TF) as trachoma prevalence decreases and to ensure accurate field TF grading in trachoma prevalence surveys. We compared photograph and field TF grading and determined the acceptability and feasibility of eyelid photography to community members and trachoma survey trainers.

**Methods:**

A total of 100 children ages 1–9 y were examined for TF in two Maasai villages in Tanzania. Two images of the right everted superior tarsal conjunctiva of each child were taken with a smartphone and a digital single-lens reflex (DSLR) camera. Two graders independently graded all photos. Focus group discussions (FGDs) were conducted with community members and Tropical Data trainers.

**Results:**

Of 391 photos, one-fifth were discarded as ungradable. Compared with field grading, photo grading consistently underdiagnosed TF. Compared with field grading, DSLR photo grading resulted in a higher prevalence and sensitivity than smartphone photo grading. FGDs indicated that communities and trainers found photography acceptable and preferred smartphones to DSLR in terms of practicalities, but image quality was of paramount importance for trainers.

**Conclusions:**

Photography is acceptable and feasible, but further work is needed to ensure high-quality images that enable accurate and consistent grading before being routinely implemented in trachoma surveys.

## Introduction

Trachoma is the leading infectious cause of blindness worldwide and is targeted for elimination as a public health problem by 2030.^1^ There are three criteria that must be met for a country to be validated by the World Health Organization (WHO) as having achieved elimination in all formerly endemic districts: a prevalence of trachomatous inflammation–follicular (TF) in children ages 1–9 y of <5%, a prevalence of trachomatous trichiasis (TT) unknown to the health system in people ≥15 y of age of <0.2% and written evidence of a strategy to identify and manage incident cases of TT.^2^ WHO recommends implementation of the SAFE (surgery, antibiotics, facial cleanliness and environmental improvement) strategy to reduce disease prevalence in districts where the elimination prevalence targets have not yet been met,^3^ with implementation of this strategy based on the prevalence of TF and TT. Reproducible clinical grading of trachoma is therefore extremely important to demonstrate achievement of the elimination of trachoma as a public health problem.

Tropical Data^4^ is a service that supports health ministries to conduct globally standardised high-quality trachoma prevalence surveys (www.tropicaldata.org) and builds upon protocols, tools and quality assurance mechanisms developed under the Global Trachoma Mapping Project (GTMP).^5,6^ A key part of the service is a stringent training program to standardise trachoma grading before surveys start. Standardisation is achieved by ensuring intergrader agreement (IGA) between trainees and grader trainers, who have in turn demonstrated good agreement with one of a small number of principal graders. Principal graders are individuals who must have achieved an IGA κ score of ≥0.8 against a GTMP/Tropical Data reference grader and participated as a trainer in at least two grader trainings.^5,7^ The Tropical Data grader training program requires two IGAs to be conducted, the first using 50 photo images that have been consensus graded as TF present or absent and the second using 50 live participants. The accuracy of live participant IGAs is optimal when 15–35 of the children have TF and a minimum of 5 (10%) must have TF for a valid IGA result to be generated.^5^ As TF becomes rarer, trainees are required to travel further in-country, or to more highly endemic countries, in order to find sufficient TF cases to conduct the field training.^8,9^ It also becomes increasingly challenging for graders to achieve good agreement with trainers in IGA tests as the prevalence of TF decreases, because the proportion of ‘borderline’ compared with ‘clear’ cases increases.^10,11^ Thus, successfully training graders to diagnose TF using existing methods will become increasingly difficult as the global prevalence of disease continues to decrease,^1^ and alternative solutions are needed to ensure high-quality grading during training and survey fieldwork.

Smartphones provide a means to equip field teams with computational capacity in a handheld, user-friendly and intuitive platform. The use of smartphones for data collection in trachoma surveys has become routine, in part due to their utilisation in the GTMP, and subsequently Tropical Data.^5,6^ The potential value of smartphone hardware and software, particularly for capture and display of photographs, has been recognised.^12–14^ More recently, the quality of photography with smartphones has become comparable to more traditional methods such as single-lens reflex (SLR) cameras or desktop computer screens.^15^ As smartphones become routinely available to programs and the population becomes increasingly familiar with them, a number of smartphone functions become valid options for deployment for trachoma programs. However, the use of photographs and smartphones must result in equivalent or better grading compared with field grading to ensure quality is maintained. It is also important to assess their acceptability and feasibility, because if they are not acceptable to graders or communities, they will not be implementable programmatically, regardless of their quality.^16^

In order to assess the potential to use photography to replace the live-participant IGA in Tropical Data training, and to replace or complement live grading in trachoma surveys, in this study we explored the agreement between field and smartphone photograph grading and the acceptability and feasibility of photography use in routine trachoma prevalence surveys. First, we aimed to understand how well-correlated field grading was with both smartphone and digital SLR (DSLR) photographs to provide recommendations on hardware, software and training needs for incorporation of photography in future trachoma prevalence surveys. Second, we aimed to improve our understanding of the acceptability and feasibility of eyelid photography to survey staff and communities through focus group discussions (FGDs). Finally, we aimed to collect feedback from survey staff on what they felt the potential additional functions were that a smartphone could provide over current practice in trachoma surveys.

## Methods

### Study design and participant selection

We employed a convergent mixed methods design, with qualitative and quantitative data collection occurring concomitantly.^17^ This study was conducted during a Tropical Data refresher ‘training of trainers’ (TOT) held in the Monduli district of Tanzania in July 2019. This region was selected due to previous survey results reporting high TF prevalence (www.trachomaatlas.org),^18^ past TOTs taking place here and having areas with sufficient TF for conducting the IGAs. Fieldwork took place in two Maasai villages, selected following preparatory TF case search visits.

To evaluate the quality of the mobile phone application images compared with field grading and SLR images, the sample size was calculated in R (R Foundation for Statistical Computing, Vienna, Austria) using a method for testing interobserver agreement with a binary outcome,^19,20^ with a power of 0.9, a threshold Kendall's W >0.9 (strong agreement), a type I error with α=0.05 and a conservative expected prevalence of 10%. A sample size of 100 participants was needed. No sample size calculation was conducted for the qualitative component of the study.

### Photo grading

In each village, the reference grader examined 50 children ages 1–9 y. Basic participant demographic data (age, gender, ethnicity, village) were collected from each study participant. All data were collected using free and open-source software Open Data Kit (ODK) (https://opendatakit.org/) and were uploaded to the secure London School of Hygiene & Tropical Medicine server.

Each participant's eyelids were everted and examined using a 2.5× magnifying loupe, with TF presence or absence diagnosed with the aid of follicle size guides.^21^ Graders used alcohol gel between participants to prevent infection cross-contamination. For each participant, the reference grader examined both eyes for trachoma using the recently updated WHO simplified grading system.^22^

Immediately after completion of the reference grader's examination of the right eye, two images of the right everted superior tarsal conjunctiva of each child were taken with a smartphone, followed by two photos with a DSLR camera. The rationale for taking two images was to maximise the likelihood of having at least one gradable image. The selection of the model and type of smartphone was the standard smartphone (Samsung Galaxy J3 2017, rear camera with 13 megapixels) used for the Tropical Data training and based on its wide availability in Tanzania. A macro lens (15× macro lens from Universal 4-in-1 lens kit; PNY Technologies, Parsippany, NJ, USA) was added to the smartphone, its selection being based on the product review and price. A familiar and existing camera open-source camera app (OpenCamera; https://opencamera.sourceforge.io/) was used to capture images of conjunctivae in the field. Due to delays receiving ethical approval, development of a smartphone app as well as testing camera settings (such as aperture, shutter speed and ISO [light sensitivity]) with macro lens were not performed before fieldwork. The standard Samsung Galaxy J3 2017 settings were therefore used, including the light-emitting diode flash. For the DSLR, a Nikon D60 was used with a 105 mm macro lens at 1:1; aperture f57 and a native flash, which is the setting recommended by the Partnership for Rapid Elimination of Trachoma (PRET) photography guide.^23^

There were two photographers (KM and CD), one for each device. The photographers did not have any trachoma experience or formal training in photography. Practice for conjunctivae photography was limited, with the photographers only able to practise taking photos of each other's eyes, and no time for field piloting, practice with children or assessment of photo-taking skills. Photo quality was determined prior to images being graded by references graders, according to the following criteria: everted eyelid visible and image not completely blurry. The field reference grader (AWS) later graded all images for the presence or absence of TF to enable the photo results to be compared with field grading. Another Tropical Data–certified reference grader (PM) also graded all images to enable comparison of photo grading results.

### Acceptability and feasibility

Four FGDs were convened to address the study objectives. The number of participants in each FGD was set to a maximum of eight, with attempts made at equal gender representation. The participant information and consent forms were provided to each participant in the appropriate language, and for the community FGD, the translator read through the entire forms in Maa before starting the FGD. The forms explained the purpose of the study, what their participation would involve and the risks, benefits and right to withdraw at any time.

One FGD was conducted with community members after the end of the IGA examination at each of the two study sites (n=8 per FGD, n=16 total). At both study sites, four males and four females consented to participate in the FGDs. The ages of FGD participants were not recorded. Community members from the communities where Tropical Data training was taking place and whose child's eyes had been photographed were eligible to take part in the FGDs using a convenience sampling method; participants were eligible to take part if they were willing and available to do so.

Two FGDs were conducted with Tropical Data trainers, one with English-speaking trainers (n=4, from Ethiopia, Nigeria, Uganda and Zambia) and the other with French-speaking trainers (n=5, from Cameroon [n=1], Chad [n=1], Côte d'Ivoire [n=2] and Guinea [n=1]). All were male and recorder trainers, except for one participant from Côte d'Ivoire who was a grader trainer. To take part in the FGDs, the trainers must have been attending the Tropical Data TOT, have experience collecting trachoma survey data and be willing and able to take part in the FGD. There were no pre-defined exclusion criteria. All participants taking part in the training were invited to join the FGDs; the individuals who took part volunteered to do so. A total of 61 participants of 26 nationalities participated in the TOT.

### Topic guides and focus group facilitation

Community FGDs were conducted in the participants’ local languages, Maa (Maasai language) and Kiswahili, with the support of a translator. FGDs with trainers were conducted in French and English, depending on the first language of the participants. Each participant was provided with the participant information and consent forms in the appropriate language, and a session during the TOT was held to explain the purpose of the study. Each FGD was facilitated by study staff to guide topics of conversation towards open questions outlined by a pre-designed topic guide (Box 1). Each individual was encouraged to speak out and was actively invited to speak where the facilitator felt their opinion had not been voiced for a particular question. Audio recordings were made of each FGD using a digital dictaphone (8 GB; Benjie, Shenzen, China) (http://www.benjie-tx.com/Digital-Voice-Recorder/286.html) then transferred to a secure computer to enable translation of the discussion by a native speaker of the FGD language into English. The audio recordings and translated transcripts were then reviewed for fidelity by native speakers of each language.

Box 1. **Topic guides for FGDs on the feasibility and acceptability of using photography in trachoma surveys**

**Community FGD topic guide**

• Has your child ever been screened for trachoma before today? Can you tell me about this?
○ Follow-up: When? Where? What was done and by whom?
• What do you think about having your child's eyelid lifted up for the examination of trachoma?
○ Follow-up: Why?
• What do you think about having a picture taken of the child's everted eyelid using the smartphone photo application for the diagnosis of trachoma?
○ Follow-up: Why?
• What do you think about having a picture taken of the child's everted eyelid using the DSLR camera for the diagnosis of trachoma?
○ Follow-up: Why?
• Would you be happy for your child to be examined again, and why, with:
○ No photo?
○ The smartphone photo application?
○ The DSLR camera?
• Could the examination process be improved?

**Trainer FGD topic guide**

• Do you have a preference between the photographs and practicalities of using a DSLR camera compared to the smartphone photo application?
○ Follow-up: Why do you prefer one over the other?
• What functions do you think a smartphone photo application could include?
○ Follow-up: Are there particular aspects, such as functionality or display that could be improved?
• Do you think the smartphone photo application could be incorporated into the Tropical Data system?
○ Follow-up: Do you think it can be incorporated into the following aspects of a trachoma survey process, and how:
  ▪ Training
  ▪ Supervision
  ▪ Confirmation of ‘borderline cases’
• Do you foresee any problems with using the smartphone photo application routinely in trachoma surveys?
○ Follow-up: Do you think mobile/internet connectivity and the location of the data storage (cloud vs hard drive) would be issues?
• Do you think consent is needed if the photos are taken for routine programmatic purposes?
○ Follow-up: Why? Do you foresee any challenges to remove the consent process?

### Statistical analysis

Trachoma prevalence and its 95% confidence intervals (CIs) were estimated from both the field examination and photo grading results using the binomial test.^24^ Kendall's coefficient of concordance (W)^25^ was used to estimate the agreement levels in the following comparisons: concordance between the two photo graders in marking DSLR and smartphone photos as ungradable; concordance between field and DSLR and smartphone photo TF grading; concordance between the two photo graders of their DSLR and smartphone photo TF grading (intergrader concordance) and concordance between the DSLR and smartphone photo TF grading by grader (intragrader concordance). We considered a Kendall's W <0.7 as low agreement, ≥0.7–<0.8 as good agreement and ≥0.8 as high agreement when comparing grades.^26^

### Acceptability and feasibility

Translated FGD transcripts were analysed using inductive content analysis to identify and aggregate common ideas, themes and concepts that were raised by FGD participants.^27^ Two reviewers independently divided the transcripts into conceptually distinct phrases and subsequently generated categories into which each phrase could be added. Both reviewers then met to agree on the categories and phrase assignments. Next, the reviewers generated a list of themes in the data from the category list. Finally, the theme list and category assignments were agreed upon between the two reviewers.

## Results

### Photo quality assessment

A total of 401 pictures were taken of 100 participants: 201 with the smartphone and 200 with the DSLR. After evaluating all photos, a total of 391 pictures (201 from the smartphone and 190 from the DSLR) were submitted to two reference graders for image grading. Ten (2.5%) photos were removed due to being of too poor quality.

The graders were unable to grade some of the photos due to image issues: grader 1 discarded 70 photos (17.9%) and grader 2 discarded 83 (21.2%) (Table 1). The most common issue found by the graders was low photo resolution, reported for 61 (87.1%) and 71 (85.5%) discarded photos by grader 1 and 2, respectively. Other issues affecting photo quality included the child moving because they did not want to have their eyelid everted for an extended period of time, the smartphone shutter speed was not fast enough, light reflections (reflections from sunlight and/or the flash) and shadows from the grader's or photographer's body or hand.

**Table 1.** TF grading results obtained from field examination and photo grading of the same eyes

Grading method	TF present	TF absent	Ungradable photo	TF prevalence^b^, % (95% CI)
Field examination^a^	22	78	-	22 (16.4 to 28.4)
Grader 1 DSLR	20	130	40 (21.1%)	13.3 (8.3 to 19.3)
Grader 2 DSLR	29	123	38 (20%)	19.1 (13.2 to 26.2)
Grader 1 phone	9	169	30 (14.4%)	5.1 (2.3 to 9.4)
Grader 2 phone	6	150	45 (22.4%)	3.8 (1.4 to 8.1)

a Field grading was conducted by photo grader 1.

^b^Photo prevalence was calculated using the number of gradable photos as the denominator.

Graders 1 and 2 discarded 30 (14.9%) and 45 (22.3%) of 201 smartphone photos, respectively (Table 1). Among the 190 DSLR photos evaluated, 40 (21.1%) and 38 (20%) pictures were discarded by grader 1 and grader 2, respectively (Table 1). Among the 100 examined eyes, 12 had both smartphone images discarded by both readers, whereas only 1 eye had both DSLR images discarded by both readers. There was good agreement between the two readers when assessing photo quality (Kendall's W=0.80, p<0.01), with agreement highest when comparing discarded photos taken with the DSLR (Table 2).

**Table 2.** Concordance between field, DSLR photo and smartphone photo TF grading

Comparison	Grader 1 vs field^a^ (Kendall's W)	Grader 2 vs field^a^ (Kendall's W)	Grader 1 vs grader 2 (Kendall's W)^b^	Grader 1 vs grader 1 (Kendall's W)^c^	Grader 2 vs grader 2 (Kendall's W)^c^
*Photo quality*					
DSLR photos	–	–	0.87 (p<0.01)	–	–
Smartphone photos	–	–	0.73 (p<0.01)	–	–
*Grading*					
DSLR photos between graders	0.61 (p<0.01)	0.65 (p<0.01)	0.88 (p<0.01)	–	–
Smartphone photos	0.64 (p<0.01)	0.54 (p<0.01)	0.72 (p<0.01)	–	–
DSLR versus smartphone photos	–	–	–	0.74 (p<0.01)	0.55 (p=0.17)

a Field grading was conducted by photo grader 1.

^b^Intergrader concordance.

^c^Intragrader concordance.

### Concordance between field and photo TF grading

During the field survey, 22 of 100 examined right eyes had TF [22% (95% CI 16.4 to 28.4)] (Table 1). Compared with field grading, photo grading consistently underdiagnosed TF. Both photo graders recorded higher TF prevalence in DSLR photos compared with smartphone photos (grader 1: 13.3% [95% CI 8.3 to 19.3] versus 5.1% [95% CI 2.3 to 9.4%]; grader 2: 19.1% [95% CI 13.2 to 26.2] versus 3.8% [95% CI 1.4 to 8.1]).

**Figure 1.**
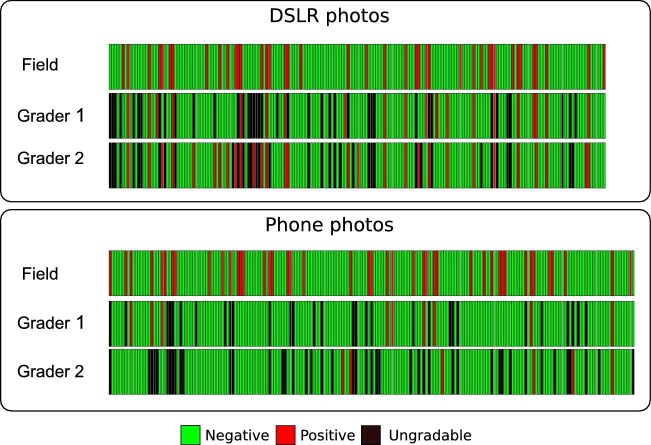
TF grading results obtained from field examination and DSLR and smartphone photos. Each bar represents a photo taken using a DSLR (n=190) or smartphone (n=201). The field bars represent the TF status of eyes in the graded pictures.

Grader 1 provided the same diagnosis as the field examination for 129 DSLR photos (67.9%, 16 TF positive and 113 TF negative) and 145 smartphone photos (72.1%, 9 TF positive and 136 TF negative), showing low agreement with field grading (Kendall's W: DSLR 0.61, p<0.01; smartphone 0.64, p<0.01) (Table 2, Figure 1). Grader 2 provided the same diagnosis as the field examination for 134 DSLR photos (70.5%, 23 TF positive and 111 TF negative) and 124 smartphone photos (61.7%, 3 TF positive and 121 TF negative), also showing a low agreement with field grading (Kendall's W: DSLR 0.65, p<0.01; smartphone 0.54, p<0.01). Both graders had high specificity but low sensitivity compared with field grading (Table 3). The sensitivity of DSLR photos was higher (grader 1, 48.5%; grader 2, 65.7%) compared with the sensitivity for smartphone photos (grader 1, 27.5%; grader 2, 9.4%).

**Table 3.** Sensitivity and specificity of photo versus field TF grading, and grader 2 compared with grader 1

Photo versus field grading				
			Sensitivity, %	Specificity, %
Grader 1 vs field^a^				
	DSLR TF present	DSLR TF absent		
Field TF present	16	17	48.5	96.6
Field TF absent	4	113		
	Smartphone TF present	Smartphone TF absent		
Field TF present	9	26	25.7	100
Field TF absent	0	136		
Grader 2 vs field				
	DSLR TF present	DSLR TF absent		
Field TF present	23	12	65.7	94.9
Field TF absent	6	111		
	Smartphone TF present	Smartphone TF absent		
Field TF present	3	29	9.4	97.5
Field TF absent	3	121		
Grader 2 vs grader 1 photo grading				
Grader 2 vs grader 1 DSLR				
	Grader 2 DSLR TF present	Grader 2 DSLR TF absent		
Grader 1 DSLR TF present	15	5	75	94.3
Grader 1 DSLR TF absent	7	116		
Grader 2 vs grader 1 smartphone				
	Grader 2 smartphone TF present	Grader 2 smartphone TF absent		
Grader 1 smartphone TF present	1	6	14.3	97.1
Grader 1 smartphone TF absent	4	136		

a Field grading was conducted by photo grader 1.

#### Intergrader photo concordance of graders

Graders provided the same diagnosis for 132 DSLR photos (64.9%, 15 TF positive and 116 TF negative) and 137 smartphone photos (68.1%, 1 TF positive and 136 TF negative) (Figure 1). Higher agreement between the two graders was reached with DSLR photos (Kendall's W=0.88, p<0.01) than with smartphone photos (Kendall's W=0.72, p<0.01). The sensitivity and specificity of grader 2’s DSLR grading compared with that of grader 1 were 75% and 94.3% (Table 3), respectively. For smartphone photos, grader 2’s sensitivity and specificity compared with grader 1’s were 14.3% and 97.1%, respectively.

#### Intragrader concordance

When the TF grading provided on DSLR and smartphone photos of the same eye was compared by grader, Grader 1 demonstrated higher intragrader concordance (Kendall's W=0.74, p<0.01) compared with that for grader 2 (Kendall's W=0.55, p=0.17) (Table 2).

### Community acceptability and feasibility

**Figure 2.**
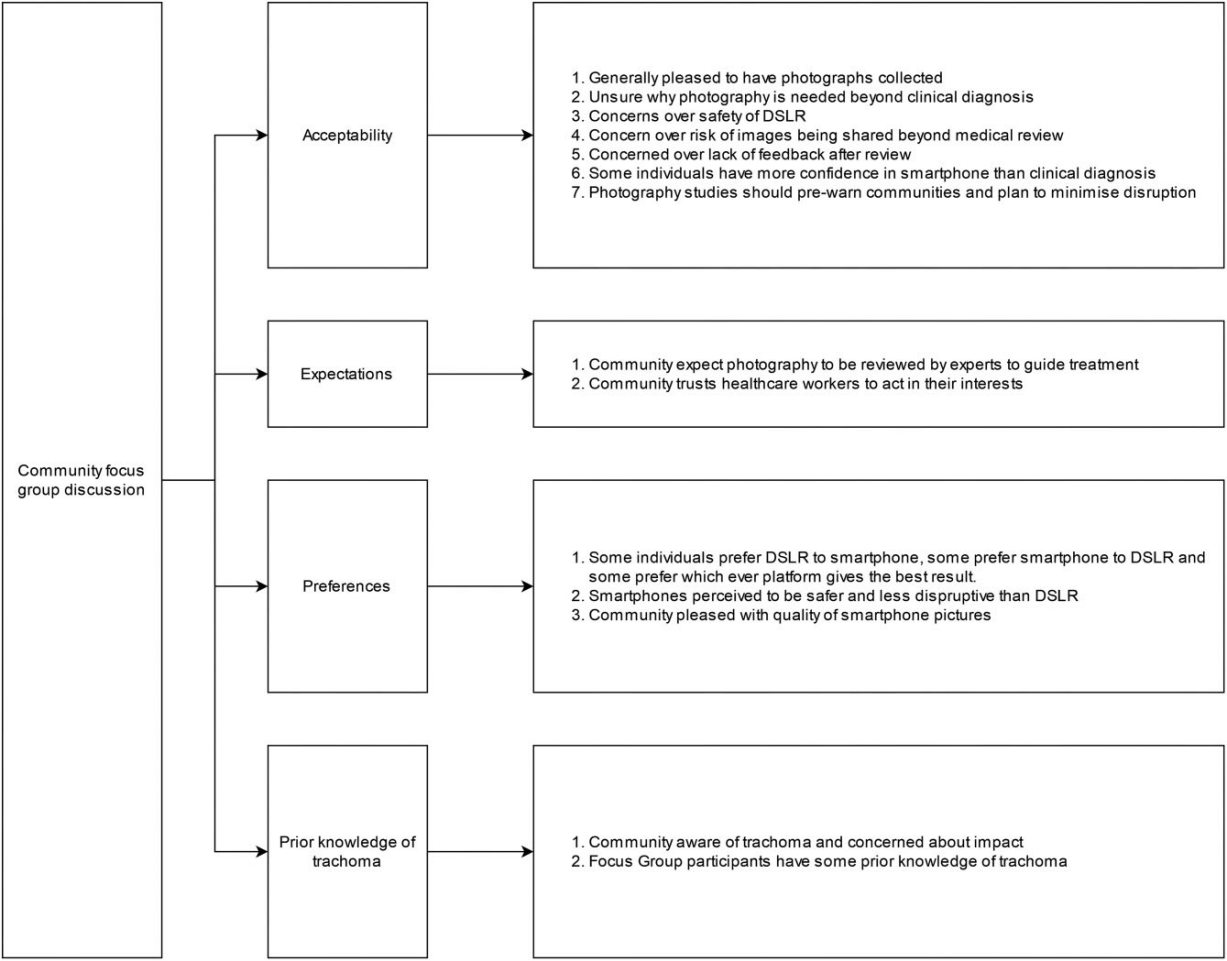
Concepts and themes emerging from FGDs with 16 adult members of two Maasai communities in northern Tanzania, July 2019.

A summary of the categories and themes that came out of the community FGDs is presented in Figure 2. There were varying levels of prior knowledge of trachoma and examinations for trachoma. Most participants were familiar with trachoma being a disease of the eye and with the process of examination. When asked about their feelings towards examination for trachoma, participants were positive, as they trusted the teams to be working to improve the health of their communities:

“We feel good because we know they want to make the children to see better and know those who are sick can be treated.”

For the same reasons, when asked about their opinion on the acceptability of taking pictures of their children's eyelids, participants were generally positive about the process and understood that high-quality images would be more likely to yield good outcomes:

“We don't feel bad when taking pictures of the eyes because it is possible you are looking for something wrong in order to control the disease.”

There were also concerns raised about the process. Clarity around schedules for children's eye examinations was sometimes lacking, leaving parents unable to plan around it:

“We need to prepare kids to have lunch if they are staying so long in the queue because it takes long for the kids to stay for the whole day without food or to have two or more days for examination so children don't stay long.”

In addition, some participants did not understand the benefit of photography in addition to clinical diagnosis:

“Why using camera, can't you use your own eyes?”

The safety of using a traditional DSLR was an element of concern, particularly the issue of the flash being scary and unsafe for young children; this was not perceived to be an issue for the smartphone:

“For the big camera, when they use the flash we think it affects the children because they have very bright light and children get scared. Smartphone is silent when taking pictures.”

The risk of images of children's eyelid being shared beyond the medical community, for purposes other than reviewing and/or confirming the trachoma diagnosis, was a potential issue:

“It is possible other people will say, where are they sending the pictures of our children?”

Participants also shared their worries about the absence or lack of feedback from the staff, following the picture being taken and sent somewhere for review:

“Sometimes some are afraid where their children's pictures are taken and they don't get feedback.”

### Trainer acceptability and feasibility

**Figure 3.**
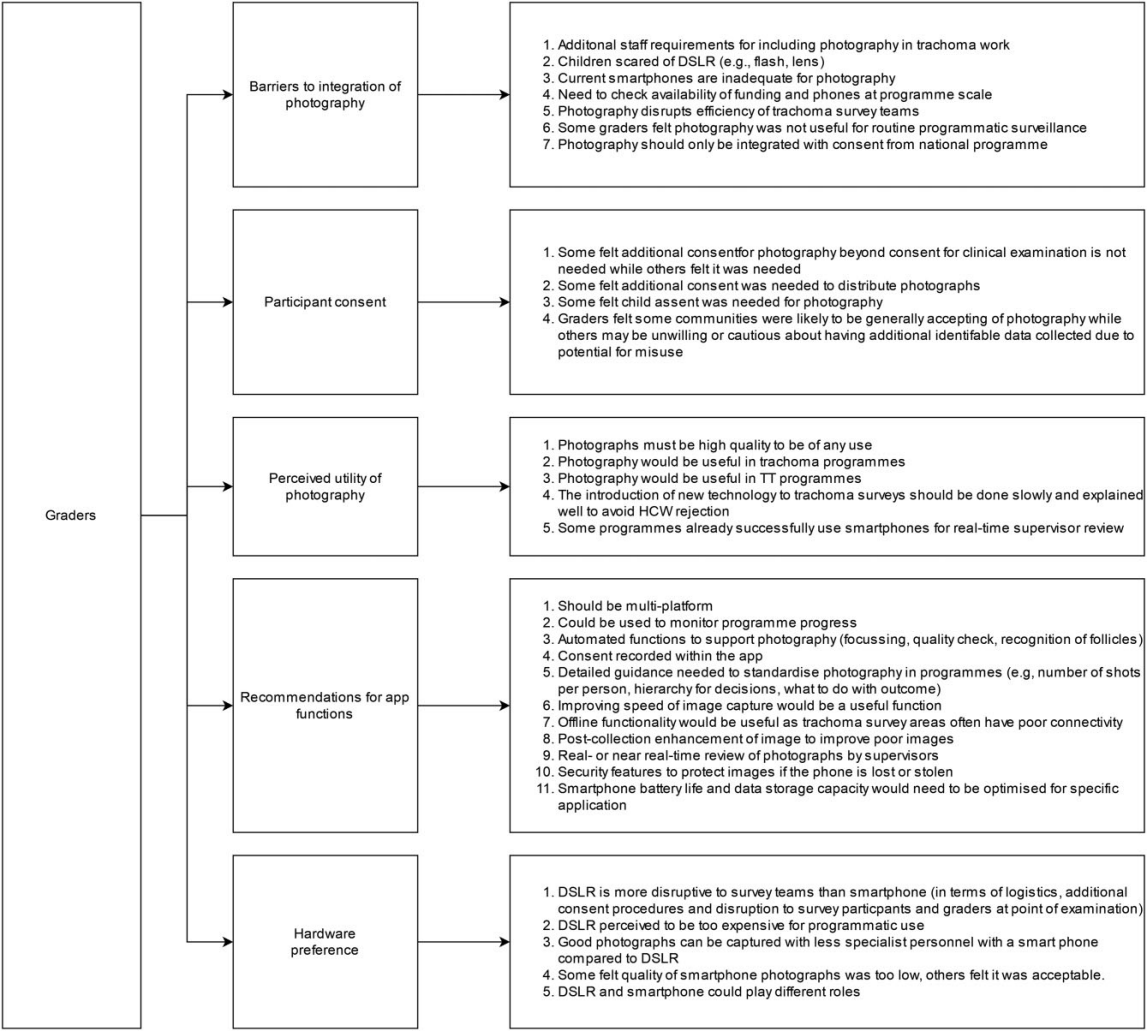
Concepts and themes emerging from FGDs with nine trainers attending a training session in northern Tanzania, July 2019. HCW: healthcare worker.

The themes and categories discussed during trainer FGDs are summarised in Figure 3. There was varying prior experience of use of photography in trachoma programs. Some trainers had never used photography in surveys, whereas others had some prior experience, e.g. for following up TT cases after surgery. The majority of trainers felt photography could play a useful role in trachoma programs:

“I think it is quite useful and good.”

The trainers felt there were a number of functions that photography could play, e.g. having a record of unusual findings in case they are questioned after the survey and building a resource library for survey debrief and future trainings:

“For instance, TT in children. Sometimes people find it, it would be useful to have pictures for additional evidence that you actually found it.”

“For training you can use the images later.”

Both FGD groups also identified real- or near-real-time review of images to verify results in borderline or unusual cases as a smartphone-specific functionality.

“If we can quickly test in the field and use Android or whatever the camera then you can send information for training graders. Using the pictures for quality control and confirmation.”

“Another advantage the smartphone will have is it will give you the ability to share your photos and get a second opinion faster than images taken from the bigger [DSLR] camera.”

When asked about which platform they thought would be more suitable for trachoma programs, trainers saw pros and cons to both platforms. The DSLR was considered to be more cumbersome and generate more pressure and stress among children being examined through fear of the camera flash, whereas the smartphone is familiar, portable and already used for trachoma survey protocols. However, the smartphone photographs were considered to be of lower quality.

“I prefer the smartphone because it is easy to use, compared to the big camera that is heavy.”

“I feel that the bigger camera needs more training and assistance and draws more attention from the community. And kids can be frightened when you are trying to change focus with bigger zoom and that can be very difficult.”

“If you would prefer having very good pictures where the eyelash touches the eyeball or something like that, then use the big camera [the DSLR].”

Trainers also identified some points for consideration if photography was to be integrated into routine survey methodologies. First, the quality of the image was an important discussion point. For some participants, the quality of the image was the single most important factor in deciding whether photography had any value. Participants acknowledged that platform choice and operator experience would be essential considerations for achieving a high-quality image.

“First of all, the picture quality, I think it's important that it's very good.”

“If the one who handles it is not a professional, no matter how good your camera might be you will still not have good pictures.”

Another point for consideration was how photographic evidence would be integrated into programmatic decision making.

“Even if you are to take pictures to take it out and confirm with colleagues and others, how much will it contribute into the work that has been done? … Do you have the opportunity to come back to change the result?”

Trainers felt that programs and donors would need to invest in higher-quality smartphones than those typically used by Tropical Data to ensure they had the appropriate camera resolution and data storage capacity to handle any potential photography components of the surveys.

“We need to focus on the type of Android phones that we are using for the research. They usually tell us to focus on getting the smaller price Androids, like 300- or 100-dollars phones. So, the quality of the Android phone also matters.”

The concept of consent was discussed in some depth. While some FGD participants felt survey participants would happily consent to photography, others recognised potential concerns among communities which should be addressed in the consent procedure:

“I don't think the individual consent should be taken. You may need to inform the person that we are taking a picture, but in most cases, people would not be worried that much if you take a picture of their eyes.”

“I think consent is important, as some people might refuse to take their pictures even though they might accept to be simply diagnosed.”

“The person could say you can take my picture but don't publish it on Facebook. So, when we get consent, we need to tell the person what we are going to do with the image.”

Recording of consent in the smartphone, indicating a specific written document might not be needed, was also suggested:

“With the smartphone, before starting the survey, we were asking for consent … there was no problem incorporating this in the smartphone.”

### Suggestions for functionality for a smartphone app for trachoma surveys

Participants were also invited to give their opinion about the features of a potential smartphone application. Common requirements were mentioned, such as the need for an application to be multiplatform and to be able to function offline, as survey areas often have poor connectivity. Optimisations for storage and battery life were also mentioned, as well as security features to protect potentially sensitive data:

“Before participating in the study, we should look at the security features for the photo that is taken… there is a component for security for the data collected on the phone, in this case photos, are secure.”

Specific features for the photograph capture module of the app were suggested, such as improving the speed of the image capture process, and a series of automated functions such as automated focus and quality check, automated follicle recognition, and a function for post-collection enhancement of poor-quality images:

“I wanted to add that the app will have to have itself some kind of quality check. If I am focusing this camera, it should indicate whether it [the image] is unacceptable.”

“The size of the follicles is subjective … an application that automatically selects the follicles that have the required size when you take the picture, to confirm, then it can help everyone.”

“When field workers send you photos that are not of good quality, you will need an application that can improve these images in order to use them.”

## Discussion

Through a convergent mixed methods approach,^17^ we have shown that photography is overall acceptable to both community members and Tropical Data trainers. With regards to converging data between the qualitative and quantitative approaches, image quality was a priority consideration in the FGDs, but the diagnostic accuracy of both DSLR and smartphone photographs compared with field grading indicated low agreement in our study, compounded by one-fifth of photos being ungradable. These results highlight the importance of considering the priorities of key stakeholders and end-users for the acceptability and feasibility of implementing a new technology and obtaining the data to demonstrate whether the innovation fulfils the stakeholders’ needs in order to inform future work towards adoption of the technology in question.

Other studies have also experienced a high proportion of ungradable photos. Naufal *et al.*’s systematic review of the utility of photography from trachoma surveys^15^ notes that only 6 of the 18 included studies reported the proportion of ungradable images, which ranged from 0.5% to 78%. Several reasons for ungradable images are proposed, including camera and photographer inadequacies. It is important to understand each of these parameters and put measures in place to mitigate them if photography is to be used as routine to support trachoma surveys.^16^ Efforts are currently in place to develop a photography training manual to address these points.

Our findings highlight that photographs had high specificity but low sensitivity compared with field grading, consistently underdiagnosing TF, with DSLR results better than smartphone results. This is consistent with the findings of others.^28^ This has important implications when considering what role photography can play in supporting prevalence surveys. Photos are already used as part of the Tropical Data grader trainings for the classroom-based IGA and for TT diagnosis training and certification via an Objective Structured Clinical Evaluation (OSCE).^7,29^ The creation of a database with a greater repository of photos, including ‘borderline’ photos representing different world regions, could help to enhance trachoma grader training.^16^ Such a database has now been created (https://trachomaphotos.tropicaldata.org/). The ability to take photos in the field can provide a supervisory opportunity to double-check field observations or provide post-survey quality checks. These were all suggestions provided by the trainers in the FGDs. However, replacing field grading completely with the use of photography will require more work, both in terms of achieving consistently high-quality images and grading with high inter- and intra-agreement. Furthermore, the logistic and cost implications of incorporating photography into survey fieldwork must be addressed.

The FGDs covered a range of topics regarding photography and provided useful guidance for future deployment of mobile technology. A number of suggestions for helpful functions of a future phone-based application were recorded, which should be incorporated into future app development pipelines. Plans should also be implemented to mitigate concerns raised about the additional disruption to the community added by using photography with trachoma surveys, the fear felt by children facing photography with a DSLR and worries about data confidentiality to ensure community trust is maintained. The community FGD participants highlighted the importance of pre-visit sensitisation, as well as feedback immediately post-examination, in order to improve participation in trachoma grading activities. The importance of good communication with communities has been noted by others^30^—if interventions are being implemented with and by communities, rather than to them, they are much more likely to be successful.

There were limitations to this study. The trainer FGD participants were all male, and only one trainer was a grader trainer. Since photography for TF grading is not routine practice in trachoma surveys, many of the trainer participants’ responses were hypothetical. As the use of photography expands (currently as operational research attached to routine surveys), future work to obtain the views of field team members with more field experience of photography in trachoma surveys would be valuable to substantiate the findings of this study. The community FGDs were culturally homogeneous from two villages. Although the community participants were recruited by the village chairman and translators, these were caregivers of the children whose eyes had been examined, and sampling bias could have been introduced. A larger sample of people with a more purposive sampling frame to ensure a variety of demographic and sociocultural backgrounds would help determine whether the findings from our study are generalisable. In addition, there may have been social desirability bias in the responses, as the FGDs were conducted by an external researcher (with support from a local translator), which may have limited strategies to avoid social desirability (such as developing a rapport with participants) and detect tendencies towards social desirability in responses (which is often based on the intuition, knowledge and experience of the participants and their culture).^31^ We also did not triangulate the data with other forms of data collection, such as in-depth interviews. A further limitation is that the age and gender of the participants were not recorded, so it was not possible to verify that the sample was evenly distributed across those variables. It was also not possible to assign FGD quotes and comments to particular individuals and their age and gender characteristics. Age and gender are major sources of variance in qualitative research and future studies should ensure different genders and ages are evenly represented.^32^

In terms of the concordance between field and photo grading, due to ethical approval delays, it was not possible to develop the smartphone app to improve photo quality or for photographers to be trained and to practice in order to improve skill. This likely contributed to the high proportion of ungradable images and low inter- and intra-agreements obtained. The DSLR camera settings were based on the PRET study parameters,^23^ whereas no standardised settings were used for the smartphone, which may help explain why smartphone images performed less well than those from the DSLR, when each was compared to field grading. We only used one type of smartphone and one DSLR camera, which limited the range of results that could be obtained. Conducting this work with a greater number of devices—especially as smartphone cameras are constantly improving and new innovations are being developed^33^—and with optimised parameters, training, certification and practice, are areas for future work. Our results showed that a small fraction of eyes could not assessed by both readers due to image quality issues. Increasing the number of photos taken might result in a higher likelihood of obtaining pictures with good quality; given that only one eye had both DSLR pictures discarded by both readers, we could suggest that taking an extra picture with a DSLR could be sufficient. However, considering that there were 12 eyes that all readers were unable to grade, the smartphone method might require more than three pictures. We believe that further investigations should be performed to estimate the optimal number of pictures for each method. Videos rather than still images might increase the likelihood that remote graders could assess the conjunctiva, but would also increase the size of files for transfer. Our images were only graded by two graders; evaluation by more graders would enable a more comprehensive assessment of between-grader photo agreement and potentially identify those with tendencies to over- or undercall TF.

The generally positive attitudes towards photography by both community members and trachoma survey trainers should encourage trachoma programs and their research partners to invest in development of smartphone-based photography platforms. This development should be combined with thorough training and certification of photographers and image graders to ensure high-quality images and results. Feedback must also be sought from stakeholders at all levels to ensure that the technology and its implementation meet the needs of trachoma programs, helping achieve the goal of global elimination of trachoma.

## Data Availability

The data underlying this article will be shared upon reasonable request to the corresponding author.
